# Aponeurosis of the levator palpebrae superioris in Chinese subjects: A live gross anatomy and cadaveric histological study

**DOI:** 10.1097/MD.0000000000004469

**Published:** 2016-08-07

**Authors:** Er Pan, Yun-Fei Nie, Zhen-Jun Wang, Li-Xia Peng, Yan-Hong Wu, Qin Li

**Affiliations:** a Southern Medical University; b Department of Plastic Surgery, Guangzhou General Hospital of Guangzhou Military Command of PLA; c Aesthetic Plastic Surgery, Hospital of the San Yet-Sun Medical University, Guangzhou; d LOMEYE Medical Beauty Clinic, Beijing, China.

**Keywords:** cadaveric histology, levator palpebrae superioris aponeurosis, Masson trichrome staining, orbital septum, ptosis, smooth muscle actin

## Abstract

An accurate understanding of the anatomy of the levator palpebrae superioris aponeurosis (LPSA) is critical for successful blepharoplasty of aponeurotic ptosis. We investigated the macroscopic and microscopic anatomy of the LPSA.

This prospective live gross anatomy study enrolled 200 adult Chinese patients with bilateral mild ptosis undergoing elective blepharoplasty. Full-thick eyelid tissues and sagittal sections from the eyelid skin to the conjunctiva were examined with Masson trichrome staining or antismooth muscle actin (SMA) immunohistochemistry.

Gross anatomy showed that the space between the superficial and deep layers of the LPSA could be accessed after incising the overlying superficial fascia, by retracting the white line. Adipose layers were clearly observed in 195 out of 200 patients with bilateral mild ptosis, among which 180 cases had the superficial layer connected to the uncoated adipose. Fifteen cases had the superficial layer connected to the smoothly coated layer, and 5 cases had the superficial layer directly connected to the deep loose fiber, almost without adipose. In previously untreated patients, the LPSA space was located beneath the intact orbital septum. In those with previous surgeries, it was beneath the superficial layer of the LPSA, underlying the destructed orbital septum. Cadaveric histology showed that the deep layer of the LPSA extended into the anterior layer of the tarsal plate and the superficial layer reflexed upward in continuity with the vertical orbital septum. An occult space existed between the 2 layers of the LPSA, with a smooth lining on the deep layer. The superficial layer of the LPSA was SMA-immunonegative but the deep layer was slightly immunopositive for SMA. An occult anatomic space exists between the superficial and deep layers of the LPSA, in proximity to the superior tarsal plate margin. Recognition of the more anatomically significant LPSA deep layer may help improve the aesthetic outcome of blepharoplasty.

## 1 Introduction

Ptosis, also called blepharoptosis, refers to abnormal drooping of the upper eyelid^[1]^ and mainly results from myogenic neuromyogenic and/or aponeurotic impairment. Myogenic causes primarily consist of congenital and acquired damage or trauma to the levator palpebrae superioris (LPS) muscle. Aponeurotic ptosis is the most frequent type of acquired ptosis due to senescence, dehiscence, or disinsertion of the LPS aponeurosis (LPSA), the tissue overlying Müller muscle and beneath the orbital septum adipose.^[2]^ Most of ptosis normally requires surgical intervention, that is, blepharoplasty, for improving vision and aesthetic appearance. Therefore, a successful blepharoplasty for treating aponeurotic ptosis depends on an accurate understanding of LPSA anatomy.

**Figure 1:**
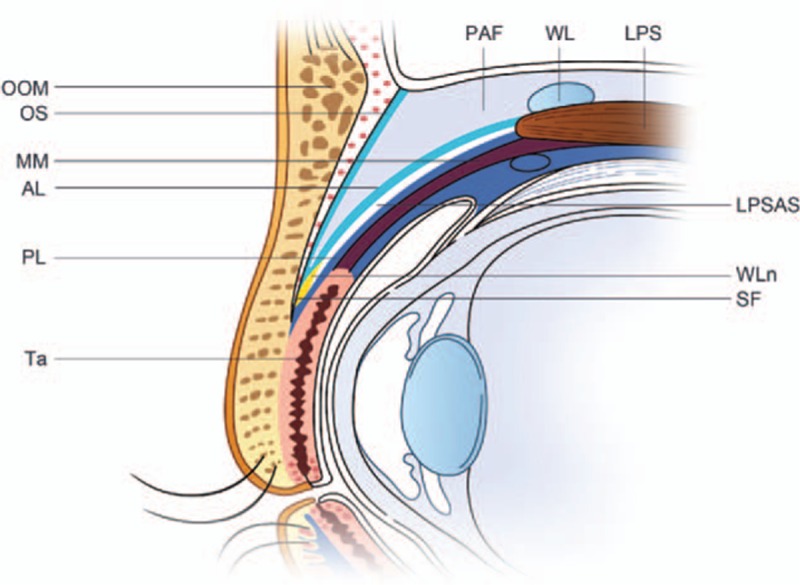
Schematic of upper eyelid anatomy. Anterior/posterior layer of the levator palpebrae superioris aponeurosis. LPS = levator palpebrae superioris, LPSAS = levator palpebrae superioris aponeurosis space, MM = Müller muscle, OOM = orbicularis oculi muscle, OS = orbital septum, PAF = preaponeurotic fat, SF = superficial fascia, Ta = tarsal plate, WL = Whitnall ligament, WLn = white line.

The LPSA is located posterior to the vertical orbital septum^[3,4]^ and the white line, a thick white connective tissue in proximity to the superior tarsal plate margin.^[5]^ The LPSA was formerly considered a uniform single-layered structure.^[2,3,5,6]^ However, subsequent studies have reported that LPSA divides anteriorly into a superficial and a deep layer, viewed from Whitnall ligament.^[7–10]^ An occult anatomic space has been observed between these 2 layers of the LPSA during upper eyelid cosmetic surgery (Fig. 1).

Precise anatomic study of the LPSA may be helpful for improving the cosmetic outcome of LPS surgical correction. This study investigated the precise macroscopic and microscopic anatomy of the LPSA in Chinese adult patients with ptosis and in cadaveric specimens.

## 2 Methods

The Institutional Review Board of Southern Medical University, Guangzhou and the Institutional Review Board of PLA Guangzhou Military Command General Hospital approved the study protocol, in accordance with the latest version of the Declaration of Helsinki. All patients voluntarily gave informed consent in writing before participation in this study.

A total of 1000 patients with ptosis were scheduled for elective eyelid cosmetic surgery at our department between January 2011 and June 2015. These patients were prospectively screened for enrollment in the present study. The inclusion criteria were ≥18 years old, with bilateral mild ptosis. Included patients were either previously untreated or treated with East Asian blepharoplasty (so-called double eyelid surgery) due to the popularity of epicanthic folds in East Asians. Patients were excluded from the study if they had any complicating ophthalmologic condition, impaired facial nerve function, or eyeball prominence, or had an overexpectation of the cosmetic outcome or rejected participation in this study.

The study finally comprised 200 patients with a mean age of 30 years (aged 18–45 years; 25 men, 175 women) eligible for gross operative anatomy examination, involving 400 upper eyelids. Among these, 30 patients (30/200, 15%) had a history of previous Eastern Asian double eyelid surgery failure that resulted in loss of orbital septum integrity.

### 2.1 Operative technique

**Figure 2:**
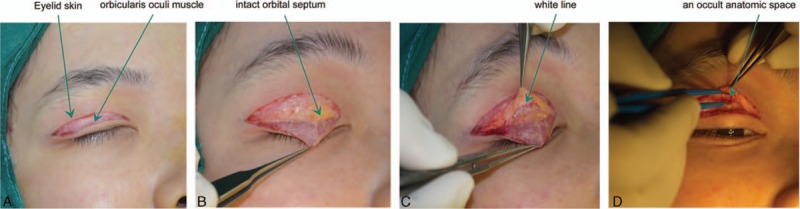
Double eyelid surgery in a previously untreated East Asian patient. (A) Incision of eyelid skin and orbicularis oculi muscle, (B) exposure of the intact orbital septum, (C) location of the white line in proximity to the superior tarsal plate margin, and (D) identification of an occult anatomic space between the superficial and deep layers of the levator palpebrae superioris aponeurosis.

Initial or second-look double eyelid surgery was planned as routine, and the eyelid skin and orbicularis oculi muscle were sequentially incised under regional anesthesia (Fig. 2A). The orbital septum was divided and exposed (Fig. 2B), and the white line, a thick connective tissue fusing the orbital septum with the LPSA, was identified in proximity to the superior tarsal plate margin (Fig. 2C). The thin fascia overlying the white line was incised, and the white line was retracted upward. An occult anatomic space was located between the superficial and deep layers of the LPSA (Fig. 2D).

### 2.2 Cadaveric histology and immunohistochemistry

The Ethics Committee at South Medical University, Guangzhou approved the use and processing of human cadaveric tissue, in accordance with China's national legal regulations regarding human tissue, organ, and cadaver use for scientific and medical purposes.

The cadaveric specimens were procured from 10 voluntary donors with a mean age of 65 years (aged 55–75 years at death; 5 men, 5 women) and fixed in 10% neutral formaldehyde at the Department of Anatomy Cadaver Dissecting Laboratory, South Medical University. Full-thickness eyelid tissues were harvested, from the superior palpebral margin to the superciliary arch. These were refixed in 4% paraformaldehyde for 24 to 36 hours and embedded (0.2 cm) in paraffin for sagittal sectioning, from the eyelid skin to the conjunctiva. Further processing was via Masson trichrome staining and antismooth muscle actin (SMA) immunohistochemistry (below). The stained sections were dehydrated through an ethanol series (0%, 50%, 75%, 85%, 95%, and 100%), permeabilized with xylene, mounted using neutral balsam, and examined under a standard light microscopic imaging system (Olympus, Tokyo, Japan) at magnifications of 40×, 100×, and 200×.

#### 2.2.1 Masson trichrome staining

The sections were dewaxed with xylene and rehydrated in a descending ethanol series (100%, 95%, 85%, 75%, 50%, and 0%). Dewaxed sections were incubated in Bouin solution at 60 °C for 1 hour, stained with hematoxylin for 3 minutes, and rinsed in 1% hydrochloride-ethanol solution for 10 seconds. The sections were subsequently rinsed in tap water for blue colorization, stained with Ponceau S fuchsin acid dye solution for 5 minutes, processed in 1% phosphomolybdic acid for 5 minutes, stained with 2% aniline blue solution for 5 to 10 minutes, and postprocessed in 1% acetic acid for 10 seconds.

#### 2.2.2 SMA immunohistochemistry

The sections were dewaxed and rehydrated as described above and rinsed in additional phosphate-balanced solution for 5 minutes. Endogenous peroxidase was blocked with 3% hydrogen peroxide for 10 minutes. The antigens were retrieved using citric acid until 8 minutes after boiling and gradually cooled to room temperature. The nonspecific binding sites were blocked with normal goat serum in a humidified incubator at room temperature for 30 minutes. The antigens were detected using primary rabbit polyclonal anti-α-SMA antibodies (1:1500; Abcam, Cambridge, MA) at 4 °C overnight. The bound primary antibodies were detected using ready-to-use horseradish peroxidase-conjugated secondary antirabbit immunoglobin G (1:200) at room temperature for 40 minutes. The secondary antibodies were colorized using 3,3′-diaminobenzidine, and the nuclei were counterstained with hematoxylin.

## 3 Results

**Figure 3:**
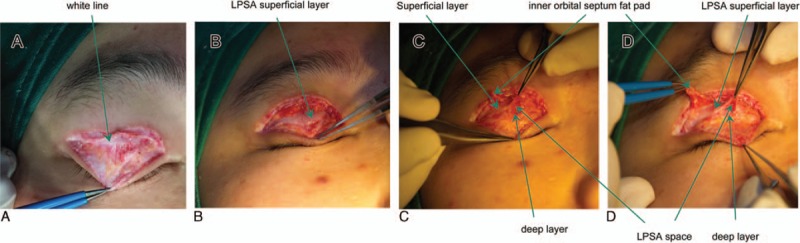
Gross anatomy of levator palpebrae superioris aponeurosis (LPSA) in a previously treated patient with a destructed orbital septum. (A) The white line in proximity to the superior tarsal plate margin, (B) the formation of the LPSA superficial layer into the orbital septum posterior wall, (C) LPSA space between the superficial and deep layers of the LPSA, and (D) the LPSA superficial and deep layers and inner orbital septum fat pad.

**Figure 4:**
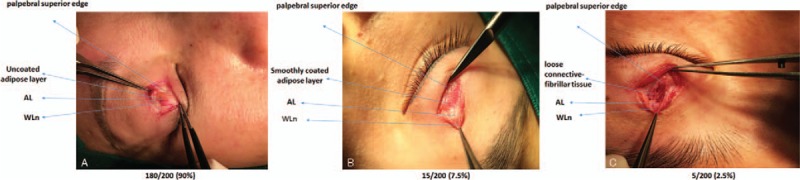
Different types of connective tissues between the levator palpebrae superioris aponeurosis superficial and deep layers. (A) The anterior layer (AL) was connected to the uncoated adipose layer (180 out of 200 patients had this mode, 90%), (B) the AL was connected to the smoothly coated adipose layer (15 out of 200 patients had this mode, 7.5%), and (C) the AL was connected to the loose connective-fibrillary tissue (5 out of 200 patients had this mode, 2.5%).

In live patients, the vertical orbital septum fused with the LPSA into the white line with a variable thickness and located in proximity to the superior tarsal plate margin (Fig. 2C). Gross examination showed that the space between the superficial and deep layers of the LPSA could be accessed after incising the overlying superficial fascia by retracting the white line (Fig. 2D). Overlying the LPSA space was the superficial layer of the LPSA, and beneath the space was the deep layer covered by adipose or fibroconnective tissue. An intact orbital septum was located above the LPSA space in previously untreated patients (Fig. 2D), and a destructed orbital septum was identified above the superficial layer in previously treated patients (Fig. 3A–D). Among the 200 patients with mild bilateral ptosis, adipose layers were clearly observed in 195 cases, among which 180 cases (Fig. 4A) had the superficial layer connected to the uncoated adipose, while 15 cases (Fig. 4B) had the superficial layer connected to the smoothly coated layer. The other 5 patients had the superficial layer directly connected to the deep loose fiber, almost without adipose (Fig. 4C).

**Figure 5:**
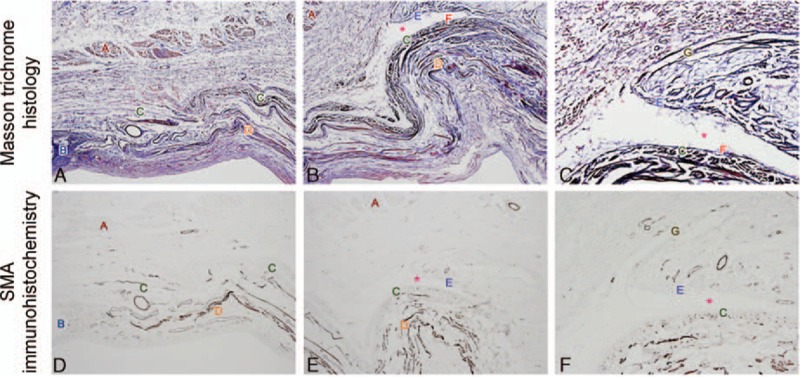
Histology and smooth muscle actin (SMA) immunohistochemistry of levator palpebrae superioris aponeurosis (LPSA). Masson trichrome histology ([A] 40×, [B] 40×, and [C] 100×) and SMA immunohistochemistry ([D] 40×, [E] 40×, and [F] 100×) of LPSA. (A) Orbicularis oculi, (B) upper edge of tarsus, (C) slightly α-SMA immunopositive muscular component in the deep layer or blue Masson staining of LPSA deep layer to tarsus, (D) dark a-SMA immunopositive or red Masson staining of Müller muscle, (E) superficial layer of LPSA, (F) space between the 2 layers with smooth surface, and (G) vertical orbital septum.

In the cadaveric study, Masson trichrome stain revealed that the deep layer of the LPSA extended onto the anterior layer of the tarsal plate (Fig. 5A), while the superficial layer reflexed upward in continuity with the vertical orbital septum (Fig. 5B). A large number of septa with a smooth lining were observed inside the LPSA space (Fig. 5C). SMA immunohistochemistry showed that the LPSA superficial layer was immunonegative to SMA and in continuity with the vertical orbital septum. The deep layer was slightly immunopositive and extended anteriorly into the tarsal plate (Fig. 5D–F), indicating that the LPSA deep layer contained some smooth muscular components.

## 4 Discussion

Müller muscle is located beneath the LPSA,^[8]^ and an occult space called the retroaponeurotic space exists between the LPSA deep layer and Müller muscle.^[11]^ Previous anatomic studies showed that the vertical orbital septum fused with the LPSA into a whitish connective tissue structure, namely, the white line, in connection with the superior tarsal plate margin.^[10–16]^ This white line in the anterior layer of the levator aponeurosis has been referred by Kakizaki et al^[10]^ as the fusion landmark of the orbital septum and LPSA. In contrast, several other groups^[2,17,18]^ reported that dissection between the orbital septum posterior wall and the LPSA exposed the LPSA space in live subjects and cadaveric specimens. However, no further studies were conducted to delineate the precise anatomic relationship of these 2 structures and the existence of the LPSA space.

The LPSA superficial layer functions as the posterior wall of the orbital septum, or an extension of the vertical orbital septum. Krohn-Hansen et al revealed that there is a dissection space in between the orbital septum and the levator, providing a minimal invasive access corridor to the structures in the upper mid-orbit.^[19]^ We here identified that an occult anatomic space exists between the superficial and deep layers of the LPSA, in proximity to the superior tarsal plate margin. Simple advancement of the LPSA deep layer could achieve a favorable cosmetic effect in patients with ptosis.^[20]^ The orbital septum was revealed to be composed of 2 layers, namely, the superficial and deep layers.^[12]^ The superficial layer, containing vertically distributed vessels, extended beneath the orbicularis oculi muscle and fused with the LPSA through loose connective tissue. The deep layer was in close proximity to the superficial layer from the originating site and reflexed into the LSPA in continuity with the LPS sheath. The continuity of the orbital septum deep layer with the LPS sheath has been reported.^[21]^ In addition, in a study of Korean cadaveric specimens, the LPS was revealed to be thoroughly enveloped by the fascial sheath^[22]^; the overlying sheath of the LPS became obviously thickened on the posterior side and formed Whitnall ligament. The LPS sheath extended forward into a thin, free margin and terminated on the superior tarsal plate margin, namely, the white line. All these results were consistent with Whitnall findings, that the thickened LPS sheath formed the superior transverse ligament, connected with the underlying LPSA, and extended into the inner orbital septum.^[23,24]^

In the present study, we found that the LPSA was composed of 2 layers and the LPS sheath completely enveloped the muscle, in continuity with the orbital septum deep layer, consistent with Whitnall description.^[23,24]^ Furthermore, our gross anatomy observations were that an occult space existed between the superficial LPSA and the deep layers of the LPSA, and the deep layer was more anatomically significant. Kakizaki et al^[8]^ reported that the LPS sheath was actually the LPSA superficial layer and did not extend into the tarsal plate anterior layer. In Kakizaki description, the LPSA superficial layer, or LPS sheath, extended into the orbital septum; however, the LPSA was inseparable, although a septum-like structure was identified underneath the LPS sheath. Studies by Anderson et al^[25,26]^ showed that the LPS extended through Whitnall ligament into the LPSA terminating at the lower 1/3 part of the superior tarsal plate, while it was generally believed that the LPS formed a broad aponeurosis forward and terminated at the lower 1/3 part of the tarsal plate as elastic fibrous aponeurosis.^[27]^ In the patient enrollment, we didn’t exclude some of the patients who had previous surgeries because we aimed to determine if the existence of an occult anatomic space between the superficial and deep layers of the LPSA is universal. This space in-between is so far not recognized well but considered *as part of LPS within the multilayers* beneath the orbital septum adipose. Thus, this structure might not be damaged in the previous surgeries. Indeed, we found the presence of this intact space also in patients who had have previous surgeries and were able to separate this structure during the operation. This result supports our hypothesis that an occult anatomic space exists between the superficial and deep layers of the LPSA. Indeed, a most recent review revealed that an adipose layer was visible after lifting up the posterior surface of the levator aponeurosis and that layer was termed as “postaponeurotic fat pad”, which is consistent with our findings.^[28]^ Therefore, in the setting of conventional blepharoplasty, folding the superficial layer alone cannot advance the deep layer, the anatomically more significant LPSA, if the occult space between these 2 layers is relatively large, which may result in an unfavorable aesthetic outcome or even operative failure.

Several previous studies reported that the LPSA was composed of 2 layers containing a muscular component, with more smooth muscle in the deep layer.^[8–10]^ However, in another study by Hwang et al^[22]^, the LPSA was revealed to be completely immunopositive to SMA but the LPS sheath was immunonegative, suggesting that the LPS sheath contained no smooth muscle fibers. In the present study, we showed that the LPSA superficial layer, as evidenced by Masson connective tissue staining, was immunonegative to SMA, while the deep layer was slightly SMA-immunopositive. Thus, it was likely that the LPSA was composed of not 2 layers, but the LPSA superficial layer is equivalent to the LPS sheath, while the LPSA deep layer is an anatomic structure mainly composed of smooth muscle. In addition, the deep layer is the anatomically significant LPSA, containing a fibromuscular component, and extending forward into the upper part of the tarsal plate. Therefore, ptosis did not worsen, but even improved, when the patient was instructed to open the eye after the superficial layer was mobilized. These findings suggest that the superficial layer is unable to elevate the eyelid but instead functions to suspend the eyeball.

There is a limitation of the present study. We have attempted to measure the exact size of the occult anatomic space. Unfortunately, it's very difficult to do so during surgeries because the margin of the space is difficult to define. We are conceiving to set up cameras to take photos from different angles and measure the 3 anatomic layers with computer aid later.

In summary, our study results showed that an occult anatomic space exists between the superficial and deep layers of the LPSA, in proximity to the superior tarsal plate margin. With preserving the orbital septum, access to the LPSA space through the white line inferior to the tarsal plate will precisely expose the LPSA deep layer that can extend onto the anterior layer of the tarsal plate. The LPSA superficial layer, containing no smooth muscle component, reflexes at the white line in continuity with the horizontal orbital septum. Recognition of the more anatomically significant LPSA deep layer may help improve the aesthetic outcome of blepharoplasty.
